# Death of the Dental Pulp by External Violence

**Published:** 1880-05

**Authors:** G. Dop, W. A. Briggs

**Affiliations:** Sacramento, from *Le Progres Dentaire.*


### ARTICLE IV.

### *Death of the Dental Pulp Provoked by External Violence.*

BY DR. G. DOP.

[Translated for the *Jairus* by W. A. Briggs, M. D., Sacramento, from *Le Progres Dentaire.*]

Patients often consult us on account of abscesses or fistulæ of the gums in connection with teeth entirely exempt from lesion. If we interrogate them as to the causes of this accident, they nearly always reply that its origin, of which they preserve no precise remembrance, was spontaneous and, generally, unaccompanied by pain.

But, as every effect pre-supposes a cause, we assert, notwithstanding the most formal denials of the patient, the pre existence of violence or external irritation ; and we are the more positive in this assertion since these accidents, when occuring in young subjects, are always met about the anterior teeth.

What is the cause of this abscess? What is the mode of its production? These are the two questions that first present themselves to the mind of the observer.

Nearly all patients, in reviewing the past, finally recollect that a blow has been experienced, and that the abscess manifested itself some months, and, in certain case, even two years afterwards.

This is not the place to discuss the anatomy of the dental pulp ; we merely observe that its structure is almost entirely *vaso nervous.* The tooth, however, has another source of vitality of which we must take cognizance ; it is called the alveolo-dental periosteum, and further on we will recognize the important function of this peridental membrane when the pulp, destroyed or calcified, is unable to maintain the physiological activity of the tooth.

We will suppose that a healthy tooth has received a blow, not violent enough, however, to produce either its fracture or its avulsion. The direction of this blow will be either perpendicular or parallel to the long axis of the tooth. What occurs in the former case? The tooth transmits the shock to the alveolar walls (anterior or posterior,) and bruising, more or less severe, of the peridental membrane may be the consequence. Inflammation of the injured part ensues. If circumscribed, this inflammation may spont neously resolve ; if not, it may invade, step by step, the entire alveolo-dental periosteum. At this moment the patient may experience some local uneasiness, of which he takes no particular notice.

The inflammation, thus abandoned to itself, by contiguity extends to the dental nerve at its entrance into the canal, and may more or less rapidly extend to the dental pulp. When the pulp is invaded, symptoms of strangulation may occur and give rise to pain that the patient will endure, nearly always attributing it to neuralgia. The duration of this condition, which, we must not forget, is established long after the occurrence of the provoking cause, is, on account of insufficient data, difficult of determination. We presume, however, that this painful period is not of long continuance, since patients do not consult us until the abscess has already been established for some time.

Take, for example, a case in which the jaw is injured by a resisting body—a table. Whether the mouth be open or closed, the various lesions will be characterized by greater or less intensity or by the seat of the abscesses that will later form either in the superior or inferior maxilla. In consequence of this shock, the concussion or "ogntre coup" of the dental pulp may determine immediate inflammation of the latter, or, indeed, the phenomena of its functional excitement.

The pulp, as we have said, is almost exclusively composed of vessels and nerves; but between these two elements is found a blastema (blasteme,) in the midst of which nucleated cells are distributed.

If we examine the physiological process, we may, by comparing with them the morbid condition of which we speak, more clearly explain the various stages that lead to the death of the pulp.

In children the pulp cavity and the dental canal are very large, and with advancing age they diminish in size in consequence of osseous deposits that form upon their walls. In old people we often find healthy teeth whose natural cavities are almost entirely obliterated. This normal phenomenon is easily explained: As the vital powers fail, we observe, in certain organs, structural alterations which consist in the formation of calcified patches of greater or less extent; the dental pulp is subject to the same influences, and these osseous deposits accumulate successively on the wall of its cavity. We also observe the same phenomenon in the case of abrasion of triturating surfaces, but here there are two different modes of the production of dentine.

3

When use has destroyed a certain thickness of the tooth, the pulp, which is thus approximated to external irritations (heat, cold, violence,) secretes layers of dentine which remove it from these irritations. As this abrasion continues, the restorative efforts increase, and the time comes, at last, when the entire pulp is replaced by dentine. Frequently, however, these processes do not develop so regularly or so favorably for the patient. In fact, these excitations, by impressing the pulp too violently, instead of being physiological, may become pathological, and reproduction no longer proceeds under the influence of the simple efforts of nature. There then occur irregular deposits, scattered without order and without utility throughout the pulp; hence, extremely painful strangulations, in whose train follows destruction of this organ.

If we bear in mind what occurs during life or in certain cases of abrasion, we will readily comprehend the formation of dental abscesses, when the shock received corresponds with the long axis of the tooth. As a result of concussion of the pulp mass, a slight inflammation develops. This may limit its influence to a simple physiological excitation of the organ. Then nodules of dentine develop irregularly, and their advent gives rise to a series of inflammatory extensions, followed always by a numerical increase of these nodules. It is then no longer possible to hope for resolution. The inflammation extends incessantly; small purulent *foci* develop, and all the parts, hitherto healthy, are soon transformed into pus.

We now find ourselves in the presence of a tooth whose closed cavity contains purulent and gaseous products. New phenomena appear; the alveolo-dental periosteum, which carpets the fundus of the alveolar cavity, inflames by contact with the products of decomposition. At the point of the root, and, as it were, inserted in its circumference, develops a small cavity, lined by pyogenic membrane, which increases in capacity as the liquid secretions accumulate. This pyogenic membrane is formed from a mass of plastic lymph exuded at the root-point in consequence of inflammation of the periosteum. This sack, constantly increasing in volume, compresses the alveolar wall, producing its absorption at a point that serves as an exit for the pus. This pus comes in contact with the deep surface of the gum, distends it, inflames it, separates it, and finally perforates and creates a fistulous opening. Such is, in a few words, the origin of abscesses, following the death of the pulp of healthy teeth subjected to external violence.

We will now briefly relate some observations that permit us to establish a rational treatment of these abscesses; a treatment whose object shall be to assure the preservation of the tooth by preventing the formation of new abscesses:

*Case.*—Our first case is that of a girl of fifteen years, who consulted us last year for the riddance of two abscesses, situated internally to the inferior maxilla, in the region of the two central incisors. After a careful examination, we were unable to discover on these teeth the least trace of lesion; neither spot, nor caries, nor crack, nor fracture, nor abrasion. Both teeth were slightly loose, and pressure, as well as percussion, produced a little pain. Thermic excitations provoked no painful sensation. Our interrogations, although as complete as possible, threw no light on the etiology of these abscesses. The patient asserted that they had no discoverable cause, and that they had arisen spontaneously. There had never been any pain, and at the time of our examination, the abscesses had already been six months in existence. As these responses were scarcely satisfactory, we besought the mother to come to our assistance, and it was only upon a pressing appeal to her memory that we obtained the desired information. The mother recollected that about two years before the simultaneous appearance of these two abscesses, her daughter had fallen upon her face and struck her jaw upon a wooden stool.

We then undertook the cure of these abscesses, that we might preserve the teeth that had given rise to them. Convinced of the death of the pulp, we had but one end to attain, the permanent removal of the source of the pus.

A pyogenic sack undeniably existed. It was necessary then, as in general surgery, to penetrate within its cavity, and by modifying its secreting surface, develop in its walls an adhesive inflammation sufficient to entail the disappearance of the abscess. For this purpose we made an opening, with appropriate instruments, upon the lingual surface of the two incisors. This opening extended to the pulp cavity, permitted the introduction to the extremity of the dental canal of fine barbed broaches intended to withdraw those remains of mortified pulp, not already disorganized by the putrid decomposition.

Then taking another smooth sound, whose free extremity was wrapped with a bit of cotton, we carried the tincture of iodine within the cavity of the sack. The small cotton tube was left in place; two days after, the abscess was notably reduced in size, and the erythemutous redness surrounding it diminished in extent. This treatment was continued for a week, and at the end of this time all traces of anterior lesions had disappeared. The patient, believing the cure complete, wished to discontinue treatment. Being skeptical, and knowing that relapses are to be feared in these cases, we insisted upon continuing the treatment for another week. Abandoning cauterizations with tincture of iodine, carbolic acid was applied by the same procedure. After four cauterizations, we considered the abscess definitively cured. One part of our task was accomplished; it remained for us to prevent relapses.

We were confronted by two teeth, whose radical canal and puly cavity communicated with the external world. If they were abandoned in this condition, the patient would be exposed to the formation of new abscesses. In fact, alimentary debris might be accidentally introduced into the cavities of the teeth, and by its decomposition provoke an inflammation about the apex of the root that would pursue the same course as the original one. Obliteration of these cavities is then indicated.

Just here we should make a remark of intrinsic importance: We have regarded the abscess as radically cured, and notwithstanding all the signs favorable to this opinion, it should be prudently controlled.

For this purpose it suffices to insert a temporary filling, which should be removed upon the first signal of alarm. A bit of cotton saturated with collodion or a solution of gutta-percha in chloroform will perfectly subserve this end. The cotton thus prepared assumes a relative hardness that prevents the imbibition of buccal fluids, or liquid aliment, and assures the impermeability of the dental canal. If, after the lapse of a certain time, fifteen days or a month according to the case, no accident supervenes, the temporary *tampon* is removed, and a permanent one introduced.

But, suppose this tentative *tampon* of some easily removable substance, has been followed by slight pain and swelling in the region of the primitive abscess; we must recur to the applications of tincture of iodine and carbolic acid.

To conclude this case, we will remark that the temporary *tampon* succeeded perfectly, and some time after a gold one was inserted in the usual manner. The cure is permanent.

We might multiply these examples, but not to exhaust your patience, we will briefly present two others that will abundantly vindicate the treatment instituted:

Second—Miss M. G., seventeen years of age. Abscess situated in the region of the left, lower central incisor. Blow from a cup eighteen months ago, abscess formed a year after the accident. Drilling into the pulp cavity; cauterization of the pyogenic membrane with carbolic acid for fifteen days. Gold filling; permanent cure.

Third.—C. L., fifteen. Palatine abscess, of fifteen days duration, in the neighborhood of the right, superior central incisor.

Six months before the appearance of this abscess, a blow had been received on the upper lip from the head of a cane. Slight fluxion, no pain; cauterization for three weeks with carbolic acid; perfect cure; gold filling after temporary *tampon*. The abscess has not reappeared.

We should remark, however, that all those teeth, whose pulp has suppurated, lose their normal color, their lustre, and turn brown.

*Conclusions.*—Every alveolar abscess, in the region of a healthy tooth, is caused by external violence.

It is curable, and treatment assures the preservation of the tooth.

This treatment consists in drilling into the pulp cavity so as to permit cauterization of the pyogenic membrane by means of small *tampons* saturated with tincture of iodine or pure carbolic acid.

Finally, the abscess being thoroughly cured, relapses are prevented by filling.—*Dental Jairus.*

